# Prehospital factors associated with ICU admission in drowning patients: a retrospective multicenter cohort study in a French coastal region

**DOI:** 10.1186/s12873-026-01481-3

**Published:** 2026-01-20

**Authors:** Quentin Mathais, Luc Jurain, Annas Sebai, Pascal Mattei, Didier Jammes, Michel Kaidomar, Gilles Kleiner, Fadi Rammal, Bruno Marquer, Pierre-Marie Bertrand, Axel Belloni, Patrick Benner, Eric Meaudre, Muriel Vergne, Jonathan Chelly, Celia Boutin

**Affiliations:** 1 Intensive Care Unit, Sainte Anne Military Teaching Hospital, Toulon, France; 2 Emergency Departement, Centre Hospitalier Avignon, Avignon, France; 3 Clinical Research Unit, Délégation à la Recherche Clinique et à l’Innovation du Groupement Hospitalier de Territoire du Var, Centre Hospitalier Intercommunal Toulon La Seyne Sur Mer, Toulon, France; 4 SAMU 83, Centre Hospitalier Intercommunal Toulon La Seyne Sur Mer, Toulon, France; 5 Emergency and Critical Care Medicine, Centre Hospitalier d’Hyères, Hyères, France; 6 Emergency Department, Centre Hospitalier Intercommunal Fréjus – Saint Raphaël, Fréjus, France; 7 Intensive Care Unit, Centre Hospitalier Intercommunal Fréjus – Saint Raphaël, Fréjus, France; 8 Emergency Department, Centre Hospitalier de Saint-Tropez, Saint-Tropez, France; 9 Emergency Department, Centre Hospitalier de la Dracénie, Draguignan, France; 10 Intensive Care Unit, Centre Hospitalier du pays d’Aix, Aix-en-Provence, France; 11 Intensive Care Unit, Cannes Hospital, Cannes, France; 12 Aix Marseille University, CNRS, EFS, ADES, Marseille, France; 13 Intensive Care Unit, Polyclinique Les Fleurs, Ollioules, France; 14 Emergency Department, Sainte Anne Military Teaching Hospital, Toulon, France; 15 Emergency Department, Centre Hospitalier Intercommunal Toulon La Seyne Sur Mer, Toulon, France; 16 Intensive Care Unit, Centre Hospitalier intercommunal Toulon La Seyne sur Mer, Toulon, France

**Keywords:** Drowning, Emergency medical services, Intensive care units, Prehospital care, Hospitalization, Risk factors

## Abstract

**Background:**

Despite its prevalence, limited data describe the European prehospital management of drowning victims outside cardiac arrest situations, and clinical decision-making remains heterogeneous across EMS systems. We aimed to describe the clinical characteristics, prehospital management, and outcomes of drowning patients, and to identify prehospital factors associated with intensive care unit (ICU) admission.

**Methods:**

We performed a retrospective multicenter study including all consecutive adult managed by the emergency medical service (EMS) call-center of the Var department (SAMU 83, France) with a “drowning” code between January 2019 and October 2022. Multivariate logistic regression analysis was used to identify prehospital factors associated with ICU admission.

**Results:**

Among 296 included patients, 232 patients (78.3%) were admitted to hospital, including 130 (43.9%) to the ICU. Thirty-eight patients (12.8%) were discharge alive without admission, and 26 (8.8%) died during prehospital management. During hospital stay, 185/232 (79.7%) required respiratory support, including 51/185 (27.6%) with mechanical ventilation. Independent factors associated with ICU admission were initial impaired consciousness (OR = 4.4; 95%CI [1.3–15.3]; *p* = 0.02), Szpilman grade ≥ 3 (OR = 29.9; 95%CI [11.0–80.9]; *p* < 0.001), and immersion duration ≥ 1 min (OR = 9.0; 95%CI [2.5–32.2]; *p* < 0.001).

**Conclusions:**

Several prehospital clinical variables were associated with ICU admission in this retrospective cohort. These findings may inform EMS triage decisions, although prospective validation is required.

**Trial registration:**

The study was prospectively registered at *Clinicaltrials.gov* on 20 February 2023 (NCT05673486).

**Supplementary Information:**

The online version contains supplementary material available at 10.1186/s12873-026-01481-3.

## Background

Drowning, defined as respiratory impairment due to submersion or immersion in liquid, ranks as the third leading cause of unintentional injury death worldwide [1]. The World Health Organization (WHO) reported 236,000 drowning deaths in 2019, although this figure is likely underestimated, particularly in low- and middle-income countries [2]. In France, drowning remains the leading cause of unintentional injury death among individuals under 25 years of age [2, 3]. Despite this burden, drowning remains a neglected public health issue, with comparatively limited research efforts and funding [4].

While advances in public education, swimming area surveillance, and prehospital resuscitation have improved outcomes over the past decades, prehospital clinical management protocols for drowning victims remains heterogeneous across Europe [5, 6]. In France and many European countries, no structured prehospital guidelines exist outside cardiac arrest (CA) management. As a result, early triage and orientation decisions in drowning cases still rely largely on clinician judgement, with limited validated prehospital criteria available in European emergency medical service (EMS) systems. The six-stage Szpilman drowning severity classification has historically been used for initial assessment and triage, but its relevance has been questioned in modern EMS practice given changes in rescue systems, increased availability of ventilatory support, and evolving patient profiles [7, 8]. Advances in critical care, including the widespread adoption of lung-protective ventilation strategies, early application of non-invasive ventilation (NIV) or high-flow nasal oxygen therapy (HFNT), and the general improvement in prehospital emergency response, have significantly modified the prognosis of drowning victims [9–11].

Several studies have investigated prognostic factors for mortality in drowning patients, yet prehospital determinants of intensive care unit (ICU) admission—a key operational outcome for EMS teams—are poorly described [12, 13]. Early identification of patients likely to require ICU resources may support EMS teams in preparing appropriate hospital orientation, mobilizing advanced life support teams, and anticipating resource allocation upon arrival.

Thus, the primary aim of this study was to describe the epidemiological characteristics, prehospital management, and outcomes of drowning patients managed by the EMS in a French region, and to identify prehospital factors associated with ICU admission.

## Materials and methods

### Study design and population

We performed a retrospective multicenter cohort study (“VAR-Drowning”), including all consecutive adult patients managed by the EMS call-center with a “drowning” code registered between January 2019 and October 2022.

Patients were included regardless of their prehospital outcome (death despite resuscitation, alive without admission, or admitted to one the nine hospitals of the region). Exclusion criteria were clinical death at initial evaluation without resuscitation attempt, diving accidents (involving traumatic mechanism, barotrauma or decompression illness), coexisting traumatic injuries, or objection from the patient or next of kin.

The study was supported by the *Centre Hospitalier Intercommunal de Toulon La Seyne-sur-Mer*, approved by the local Ethics Committee on 5 January 2023 (IRB number 00012962) and registered at *ClinicalTrials.gov* on 20 February 2023 (NCT05751655). Patient or next of kin information and data use adhered to French legislation and the Helsinki Declaration of 1975. This study follows the STROBE guidelines for reporting observational studies [14].

### Study setting

The Var department is a Mediterranean coastal area with extensive beaches, intense seasonal tourism, and a high incidence of water-related emergencies. The local EMS system (SAMU 83) includes physician-staffed units, paramedical teams, and fire department first responders, and covers a population ranging from 1 to 9 million according to seasonal tourist fluctuations. Transport modalities include ground ambulances and, when required, helicopter medical evacuation; no dedicated maritime ambulance system is operated. This organizational context influences prehospital management pathways and the operational relevance of identifying severe drowning cases early.

### Data collection

Data collection followed the 2015 Utstein-style template [15]. Prehospital data were extracted from the EMS database and the prehospital emergency teams reports, including patient characteristics, clinical parameters, Szpilman classification, prehospital management details, and hospital destination. Hospital data were obtained from medical records of the nine hospitals where patients were admitted. They covered respiratory support modalities (oxygen therapy, HFNT, NIV, mechanical ventilation (MV)), antibiotic prophylaxis, ICU and hospital length of stay, and vital status at discharge. Environmental factors such as weather conditions and water type were also documented. Water temperature was assumed non-freezing based on the Utstein criteria.

The six-stage Szpilman drowning severity classification, widely used in European EMS systems for initial triage, was used to assess initial pulmonary and hemodynamic impairment [7]: No symptoms (stage 0); Cough, normal pulmonary auscultation (stage 1); Cough with rales in some fields (stage 2); Acute pulmonary edema, normal blood pressure (stage 3); Acute pulmonary edema with hypotension or shock (stage 4); Respiratory arrest and/or coma (stage 5); Cardiac arrest (stage 6).

### Statistical analysis

Categorical variables are expressed as n (%) and compared using the Chi-square test. Continuous variables were examined using visual inspection of their distribution (histograms and Q–Q plots). Variables showing approximately symmetrical distributions are presented as mean ± standard deviation (SD). Time-related variables (such as prehospital delays and durations of respiratory support), which typically exhibit skewed distributions, are presented as median with interquartile range (IQR). Continuous variables were compared using the Wilcoxon test. Univariate logistic regression identified potential factors for ICU admission (using Wald test). Variables with a p-value < 0.2 in univariate analysis were included in the multivariate analysis using backward stepwise selection procedure to identify independent factors associated with ICU admission. Odds ratios (ORs) were expressed with their 95% confidence intervals (CIs). Statistical significance was set at *p* < 0.05. Analyses were performed using SAS Studio version 3.8 (SAS institute, USA).

## Results

**Fig. 1:**
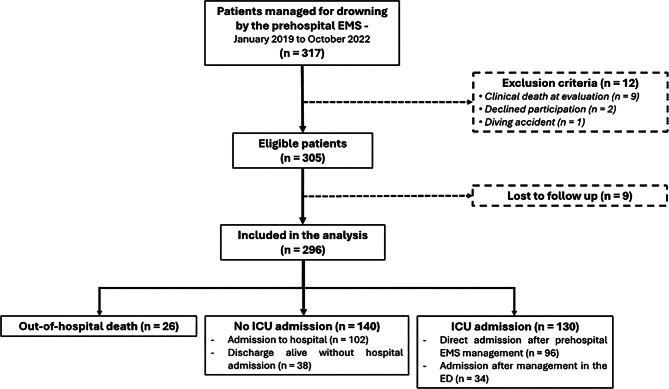
Flow chart of the VAR-drowning study (EMS: emergency medical service; ICU: intensive care unit; ED: emergency department)

During the study period, 317 drownings were recorded, and 296 patients were included in the final analysis (Fig. 1).

### Patients’ and drowning events’ characteristics (Table 1)

**Table 1:** Baseline and drowning characteristics of the 296 included patients

Variable	Available data	Category	Results ^a^
Age – years	*296 /296*		69 (± 17)
Sex	*296 /296*	Male	162 (54.7)
		Female	134 (45.3)
Chronic comorbidities	*266 /296*	Cardiovascular	97 (36.4)
		Psychiatric	37 (13.9)
		Neurological	37 (13.9)
		Respiratory	17 (6.4)
		Epilepsy or syncope	5 (1.9)
		Other	70 (26.3)
		None	63 (23.7)
Precipitating event	*150 /296*	Exhaustion	46 (30.6)
		Pre-syncope	42 (28.0)
		Trauma	26 (17.3)
		Toxic	12 (8.0)
		Syncope	7 (4.7)
		Breath holding	6 (4.0)
		Boat falling	4 (2.7)
		Suicide	4 (2.7)
		Seizure	3 (2.0)
Time of day	*296 /296*	08:00 am to 1:00 pm	97 (32.8)
		1:00 pm to 7:00 pm	174 (58.8)
		7:00 pm to 08:00 am	25 (8.4)
Location of drowning	*296 /296*	Seaside (< 300 m)	241 (81.4)
		Swimming pool	36 (12.2)
		Sea (> 300 m)	4 (1.4)
		Lake or river	9 (3.0)
		Harbor	6 (2.0)
Witnessed drowning	*290 /296*	Lifeguard	155 (53.4)
		Public	135 (46.6)
Lifeguards-supervised area	*294 /296*	Yes	193 (65.6)
		No	101 (34.4)
Duration of immersion	*234 /296*	< 1 min	173 (74.2)
		[1–5[ min	33 (13.7)
		[5–10] min	9 (3.9)
		> 10 min	19 (8.2)
CA occurrence	*296 /296*	Yes	90 (30.4)
		No	206 (69.6)
Bystander CPR	*90 /90*	Yes	86 (95.5)
		No	4 (4.5)

Categorical variables are expressed as n (%); continuous variables are expressed as mean (± standard deviation)

CA: cardiac arrest; CPR: cardiopulmonary resuscitation

a) For each variable, percentages or means were calculated based on the available corresponding data

Mean age was 69 ± 17 years, with cardiovascular diseases as the most frequent comorbidity (36.4%). Most incidents occurred during the daytime (91.6%), near beaches (81.4%), and in lifeguard-supervised areas (65.6%). A short immersion duration (≤ 1 min) was reported in 173 patients (74.2%). Seasonal variations showed a peak during summer months with 238 victims (80.4%) between June and August (Online supplement – 1). Overall mortality was 18.6% (Online supplement – 2).

### Prehospital management (Table 2)

**Table 2:** Prehospital clinical parameters and management in the overall cohort and according to Szpilman classification

Variable	Available data	Overall cohort *N* = 296	Grade 1 *N* = 59	Grade 2 *N* = 46	Grade 3 *N* = 96	Grade 4 *N* = 5	Grade 5 *N* = 25	Grade 6 *N* = 65
**Prehospital team**	*296 /296*							
Medical team		183 (61.8)	7 (11.9)	7 (15.2)	75 (78.1)	5 (100)	24 (96.0)	65 (100)
Paramedical team		98 (33.1)	37 (62.7)	39 (84.8)	21 (21.9)	0 (0)	1 (4.0)	0 (0)
Fire department		15 (5.1)	15 (25.4)	0 (0)	0 (0)	0 (0)	0 (0)	0 (0)
**Initial vital parameters**								
AVPU score	*295 /296*							
Alert		214 (72.3)	58 (98.3)	45 (97.8)	85 (88.5)	4 (80.0)	19 (76.0)	3 (4.6)
Verbal		11 (3.7)	1 (1.7)	1 (2.2)	7 (7.3)	1 (20.0)	1 (4.0)	0 (0)
Pain		3 (1.0)	0 (0)	0 (0)	2 (2.1)	0 (0)	1 (4.0)	0 (0)
Unresponsive		67 (22.6)	0 (0)	0 (0)	2 (2.1)	0 (0)	3 (12.0)	62 (95.4)
HR – bpm	*222 /296*	93 ± 23	89 ± 16	100 ± 23	95 ± 23	69 ± 26	104 ± 26	76 ± 24
SBP – mmHg	*228 /296*	139 ± 28	139 ± 23	141 ± 22	145 ± 30	79 ± 6	142 ± 25	125 ± 34
Temperature – °C	*77 /296*	34.6 ± 2.4	35.8 ± 1.4	35.9 ± 1.5	36.1 ± 1.1	36.9 ± 0	35.6 ± 0.7	32 ± 3.1
SpO_2_ – %	*236 /296*	85 ± 12	97 ± 4	88 ± 8	79 ± 11	80 ± 11	80 ± 16	87 ± 12
RR – cycle per min	*157 /296*	25 ± 11	20.6 ± 5.9	24.9 ± 7.4	28.1 ± 7.9	27 ± 11.3	NA	NA
**First aid by witnesses**	*295 /296*							
None		114 (38.5)	49 (83.1)	18 (39.1)	43 (44.8)	1 (20.0)	0 (0)	3 (4.6)
Bystander CPR		87 (29.4)	0 (0)	0 (0)	1 (1.0)	0 (0)	24 (96.0)	62 (95.4)
Oxygen therapy		84 (28.4)	9 (15.3)	25 (54.3)	46 (47.9)	3 (60.0)	1 (4.0)	0 (0)
Recovery position		10 (3.4)	0 (0)	3 (6.5)	6 (6.2)	1 (20.0)	0 (0)	0 (0)
**Medical management of CA**	*289 /296*							
Defibrillation		12 (4.1)	0 (0)	0 (0)	0 (0)	0 (0)	0 (0)	12 (18.5)
Epinephrine – mg		NA	0 (0)	0 (0)	0 (0)	0 (0)	0 (0)	3 ± 7
CPR duration – min		NA	NA	NA	NA	NA	3 ± 2	30 ± 15
**Respiratory support**	*283 /296*							
Oxygen therapy		86 (29.1)	0 (0)	43 (93.5)	38 (39.6)	1 (20.0)	2 (8.0)	2 (3.1)
NIV		78 (26.4)	0 (0)	0 (0)	54 (56.3)	3 (80.0)	17 (68.0)	4 (6.2)
MV		56 (18.9)	0 (0)	0 (0)	2 (2.1)	1 (20.0)	5 (20.0)	48 (73.8)
No respiratory support		63 (21.2)	58 (98.3)	3 (6.5)	1 (1.0)	0 (0)	1 (4.0)	0 (0)
**Prehospital outcome**	*296 /296*							
Hospital admission		232 (78.4)	22 (37.3)	45 (97.8)	96 (100)	5 (100)	25 (100)	39 (60.0)
Discharge without admission		38 (12.8)	37 (62.7)	1 (2.2)	0 (0)	0 (0)	0 (0)	0 (0)
Prehospital death		26 (8.8)	0 (0)	0 (0)	0 (0)	0 (0)	0 (0)	26 (40.0)
Total prehospital time * – min	*258 /296*	83 [60– 109]	53 [45–66]	64 [55–88]	83 [64–114]	97 [78–123]	96 [78–123]	106 [88–135]

Categorical variables are expressed as n (%); continuous variables are expressed as mean (± standard deviation); Time-related variables (*) are expressed as median [interquartile range]. AVPU: Alert, Verbal, Pain, Unresponsive; HR: heart rate; SBP: systolic blood pressure; SpO_2_: pulse oximetry saturation; RR: respiratory rate; CPR: cardiopulmonary resuscitation; CA: cardiac arrest; NIV: noninvasive ventilation; MV: invasive mechanical ventilation

Most patients (61.8%) were managed by a medical team, particularly severe cases. Less severe drownings were managed by paramedical or fire department teams (33.1% and 5.1%, respectively). Regarding AVPU scoring, 67 patients (22.6%) were unresponsive, and cardiopulmonary resuscitation was initiated by bystanders in 87 patients (29.4%). Hospital admission occurred in 78.4% of patients, whereas 38 (12.8%) were discharge alive without admission and 26 died (8.8%) during prehospital management.

### In-hospital management (Table 3)

**Table 3:** Respiratory support and outcome of the 232 patients admitted to the hospital, in the overall cohort, and according to Szpilman classification

Variable	Available data	Overall Cohort *N* = 232	Grade 1 *N* = 22	Grade 2 *N* = 45	Grade 3 *N* = 96	Grade 4 *N* = 5	Grade 5 *N* = 25	Grade 6 *N* = 39
**Maximum respiratory support**	*232 /232*							
No respiratory support		47 (20.3)	22 (100)	22 (48.9)	1 (1.0)	1 (20.0)	1 (4.0)	0 (0)
Oxygen therapy		41 (17.7)	0 (0)	23 (51.1)	17 (17.7)	0 (0)	1 (4.0)	0 (0)
HFNT		5 (2.2)	0 (0)	0 (0)	4 (4.2)	0 (0)	0 (0)	1 (2.6)
NIV		41 (17.7)	0 (0)	0 (0)	34 (35.4)	1 (20.0)	5 (20.0)	1 (2.6)
NIV + HFNT		47 (20.3)	0 (0)	0 (0)	33 (34.4)	1 (20.0)	11 (44.0)	2 (5.1)
MV		51 (22.0)	0 (0)	0 (0)	7 (7.3)	2 (40.0)	7 (28.0)	35 (89.7)
**Timing of intubation**	*232 /232*	51 (22.0)	0 (0)	0 (0)	7 (7.3)	2 (40.0)	7 (28.0)	35 (89.7)
Out-of-hospital		44 (19.0)	0 (0)	0 (0)	3 (3.1)	1 (20.0)	6 (24.0)	34 (87.2)
In-hospital		7 (3.0)	0 (0)	0 (0)	4 (4.2)	1 (20.0)	1 (4.0)	1 (1.6)
**MV duration** – days	*232 /232*	4 [2–8]	0 [0–0]	0 [0–0]	1 [1–5]	2 [1–3]	3 [1–10]	5 [3–9]
**Number of NIV sessions**	*230 /232*							
0		137 (59.1)	22 (100)	45 (100)	28 (29.2)	2 (40.0)	6 (24.0)	34 (87.2)
[1–5]		83 (35.8)	0	0	60 (62.5)	3 (60.0)	16 (64.0)	4 (10.3)
[6–10]		9 (3.9)	0	0	8 (8.3)	0 (0)	0 (0)	1 (2.6)
**HFNT duration** – days	*231 /232*	1 [1–2]	0 [0–0]	0 [0–0]	1 [1–2]	1 [1–1]	1 [1–2]	2 [1–6]
**Oxygen therapy duration** – days	*228 /232*	1 [1–2]	0 [0–0]	1 [1–1]	1 [1–2]	1 [1–5]	1 [1–3]	2 [1–4]
**Antibiotic administration**	*219 /232*	156 (67.2)	3 (13.6)	18 (40.0)	78 (81.3)	4 (80.0)	19 (76.0)	35 (89.7)
**In-hospital mortality**	*232 /232*	29 (12.5)	0 (0)	0 (0)	1 (1)	0 (0)	1 (4.0)	27 (69.2)
**ICU LOS** – days	*232 /232*	1 [0–3]	0 [0–0]	0 [0–1]	2 [1–3]	2 [1–5]	2 [1–5]	5 [2–11]
**Hospital LOS** – days	*223 /232*	2 [1–6]	0 [0–0]	1 [0–1]	3 [1–5]	4 [1–10]	5 [3–10]	7 [3–16]

Categorical variables are expressed as n (%); continuous variables are expressed as mean (± standard deviation); Time-related variables (*) are expressed as median [interquartile range]

HFNT: high-flow nasal therapy; NIV: noninvasive ventilation; MV: invasive mechanical ventilation; ICU: intensive care unit; LOS: length of stay

Of patients admitted to hospital, 130/232 (56%) were admitted to ICU, including 96/130 (73.8%) who were directly admitted after their prehospital management. Among the remaining 136/232 patients (58.6%) who were admitted to an emergency department, 8/136 (5.9%) required an interhospital transfer.

During their hospital stay, 185 patients (79.7%) underwent respiratory support, including 51 (22.0%) with MV, and 7/51 (13.7%) who were intubated in the hospital. The hospital mortality rate was 12.5%.

### Factors associated with ICU admission

After univariate analysis, several prehospital variables were significantly associated with ICU admission. These included age ≥ 75 years (*p* = 0.012), an initial respiratory rate ≥ 20 breaths per minute (*p* = 0.013), the presence of at least one chronic comorbidity (*p* < 0.001), the absence of initial management by a professional first responder (*p* = 0.048), impaired consciousness on the AVPU scale (classified as V, P, or U), initial pulse blood oxygen saturation (SpO_2_) < 94%, immersion duration ≥ 1 min, and a Szpilman grade ≥ 3 (both *p* < 0.001) (Online Supplement – 3). Multivariate analysis identified three independent prehospital factors associated with ICU admission: V, P, or U classification in the AVPU score (OR = 4.37 [1.25–15.30]; *p* = 0.021), Szpilman grade ≥ 3 (OR = 29.85 [11.01–80.91]; *p* < 0.001), and immersion duration ≥ 1 min (OR = 8.97 [2.50–32.19]; *p* < 0.001) (Online Supplement – 4).

## Discussion

In this multicenter retrospective cohort study of 296 adult drowning victims managed by the EMS in a French coastal region, we identified several key findings. First, we provided a detailed description of patient and event characteristics, including prehospital and hospital management – with a particular focus on ventilatory support strategies – and outcomes. Second, we identified three prehospital factors associated with ICU admission: initial impaired consciousness, Szpilman grade ≥ 3, and immersion duration ≥ 1 min.

The demographic profile of our cohort is consistent with previous French studies [2, 8, 16], although we observed a slightly older population, with a higher prevalence of cardiovascular comorbidities. This likely reflects the ageing demographics of the Var region and emphasizes the need for tailored preventive strategies in vulnerable populations. Drowning incidents predominantly occurred in coastal waters, often in lifeguard-supervised areas, highlighting the persistent risks despite supervision. A significant proportion of incidents also involved difficult-to-access beaches, where supervision levels were lower, potentially delaying early rescue and resuscitation efforts [8]. Concerning respiratory support, our study highlights the frequent use of NIV and HFNT in both prehospital and hospital settings among drowning victims presenting with respiratory distress (Szpilman grades 3 to 5). This pattern of practice is consistent with recent reports describing increased use of non-invasive respiratory support in drowning-related acute lung injury [10, 16–18]. Although these modalities are commonly used in clinical practice, current evidence does not yet al.low conclusions regarding their comparative effectiveness, and no formal guidelines recommend NIV or HFNT for drowning management [19]. Our findings therefore illustrate contemporary practice rather than demonstrating benefit. Ongoing research, including a French randomized controlled trial (NCT06183827), will be essential to clarify the role of non-invasive respiratory support in this setting. Interestingly, we observed a very low proportion of Szpilman grade 4 patients, consistent with recent reports suggesting that pure hemodynamic failure without respiratory arrest is rare in drowning [8, 20–22]. Moreover, among Szpilman grade 5 patients (respiratory arrest), the majority (76%) were classified as alert upon prehospital neurological assessment. This observation should be interpreted cautiously as it reflects documentation at the time of EMS evaluation rather than immediate post-rescue status. These findings highlight limitations in the Szpilman classification, notably regarding its prehospital applicability and sensitivity to early changes [7]. Alternative classification, such as the WHO non-fatal drowning framework, may offer more reliable stratification based on respiratory impairment severity [23]. Overall mortality in our cohort was 18.6%, higher than Szpilman’s original report [7] but comparable to more recent cohort [8]. The reduction in mortality for Szpilman grades 3–5 over the past decades likely reflects improvements in early rescue, resuscitation techniques, and critical care management [8, 20]. Conversely, mortality for Szpilman grade 6 patients (CA) remains high and unchanged, emphasizing the need for robust public health strategies promoting water safety education and widespread bystander cardiopulmonary resuscitation training [6]. Finally, our multivariate analysis identified three independent factors associated with ICU admission, without implying causal prediction. The Szpilman classification, despite its limitations, thus retains clinical utility for identifying high-risk patients. Impaired consciousness is a well-established indicator of poor outcomes in both drowning and general trauma populations [24, 25]. The AVPU scale, although less detailed than the Glasgow Coma Scale (GCS), provides a simple and reliable tool for prehospital triage. Finally, immersion duration ≥ 1 min, although based on subjective estimates, was strongly and significantly associated with severity, consistent with prior studies linking prolonged hypoxia to worse outcome [12]. Early identification of these characteristics in the prehospital phase may help EMS clinicians anticipate appropriate destination decisions and improve transition of care, although validation in prospective studies is necessary.

## Limitations

Several limitations must be acknowledged. First, although multicentric, the study was conducted within a single French coastal EMS system, which may limit generalizability; however, demographic and clinical characteristics were consistent with recent national and international data [2, 7, 13]. Second, as a retrospective study, missing data (~ 9.9%) may have affected the precision of analyses; in accordance with STROBE recommendations, no imputation was performed [14]. Consequently, some clinically relevant variables (e.g., respiratory rate) could not be included in the multivariable model, and others such as comorbidities or initial SpO₂ may not have emerged as independent associated factors due to missingness, limited sample size, or collinearity with stronger severity indicators (AVPU, Szpilman classification). Third, case identification based solely on the EMS “drowning” code may have missed atypical presentations, although this is unlikely to have affected the identification of factors associated with ICU admission. Fourth, neurological assessment relied on the AVPU scale, as the Glasgow Coma Scale was not routinely documented; EMS response times, water temperature, and precise timing of rescue efforts were also unavailable. Nevertheless, the AVPU scale has been shown to correlate well with GCS scores in critical care triage and remains practical for use by first responders in dynamic and time-sensitive environments [24, 25]. Fifth, the exclusion of pediatric patients limits applicability to adult drowning, although this ensured a more homogeneous study population. Finally, the study identifies associations rather than predictive relationships. ICU admission is an operational endpoint influenced by organizational and human factors in addition to clinical severity, and the number of deaths in our cohort was insufficient to allow meaningful analysis of mortality. While several studies have already addressed prognostic factors for mortality among critically ill drowning patients admitted to the ICU, data specifically aimed at guiding prehospital decision-making remain relatively limited [8, 10, 12, 13].

## Conclusions

This retrospective multicenter study describes the prehospital and hospital management of drowning victims in a French coastal region and identifies several prehospital clinical variables associated with ICU admission. These severity indicators may help support EMS triage and early orientation, although prospective studies and larger registries are needed to validate their operational relevance.

## Supplementary Information

Below is the link to the electronic supplementary material.

Supplementary Material 1

## Data Availability

Research data and other material (including the full protocol) will be made available to the scientific community, immediately upon publication, with as few restrictions as possible. All requests should be submitted to the corresponding author who will review with the other investigators for consideration. A data use agreement will be required before the release of participant data and institutional review board approval as appropriate.

## Declarations

### Patient and public involvement

Patients and/or the public were not involved in the design, or conduct, or reporting, or dissemination plans of this research.

## Abbreviations

AVPU: Alert
CA: Cardiac arrest
CI: Confidence interval
EMS: Emergency medical service
GCS: Glasgow coma scale
HFNT: High-flow nasal oxygen therapy
ICU: Intensive care unit
MV: Mechanical ventilation
NIV: Non-invasive ventilation
OR: Odds ratio
SpO_2_: Pulse blood oxygen saturation
WHO: World Health Organization
