# Supplementary material for: Prehospital factors associated with ICU admission in drowning patients: a retrospective multicenter cohort study in a French coastal region
Source: BMC Emerg Med. 2026 Jan 20;26:54. doi: 10.1186/s12873-026-01481-3 (PMC12903680; doi:10.1186/s12873-026-01481-3)
Supplement: Supplementary file 1 — Supplementary Material 1 [file 12873_2026_1481_MOESM1_ESM.docx]

**Prehospital predictors associated with ICU admission in drowning patients: a multicenter cohort study in a French coastal region.**

Quentin Mathais, Luc Jurain, Annas Sebai, Pascal Mattei, Didier Jammes, Michel Kaidomar, Gilles Kleiner, Fadi Rammal, Bruno Marquer, Pierre-Marie Bertrand, Axel Belloni, Patrick Benner, Eric Meaudre, Muriel Vergne, Jonathan Chelly, Celia Boutin.

**ONLINE SUPPLEMENTAL CONTEN**


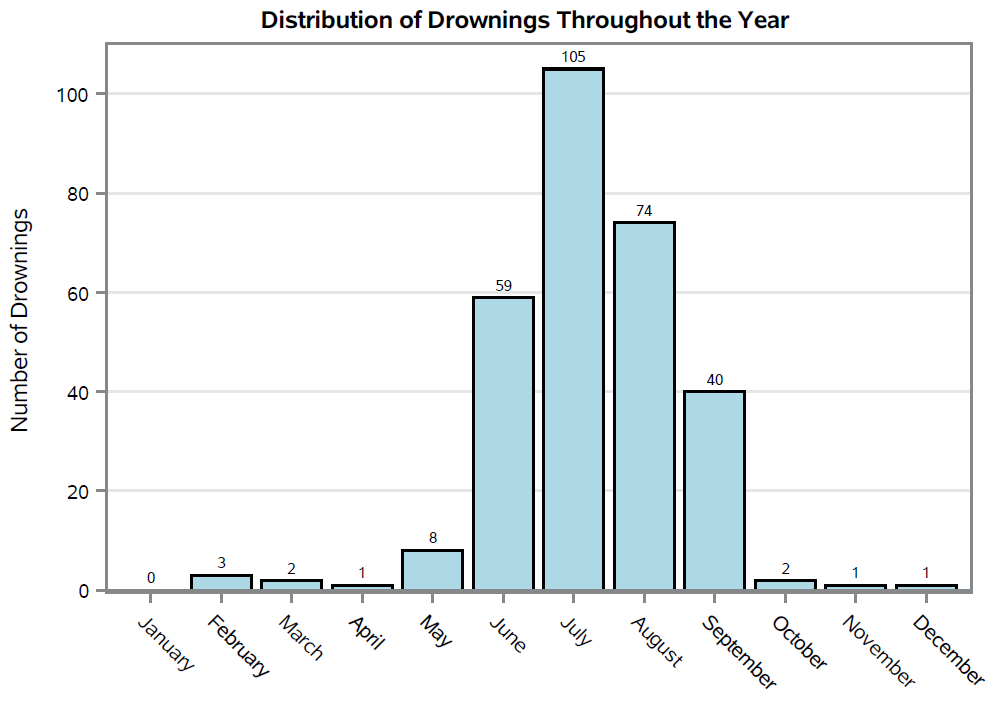
**Online supplement – 1.** Seasonal variation among the 296 reported drowning during the study period.


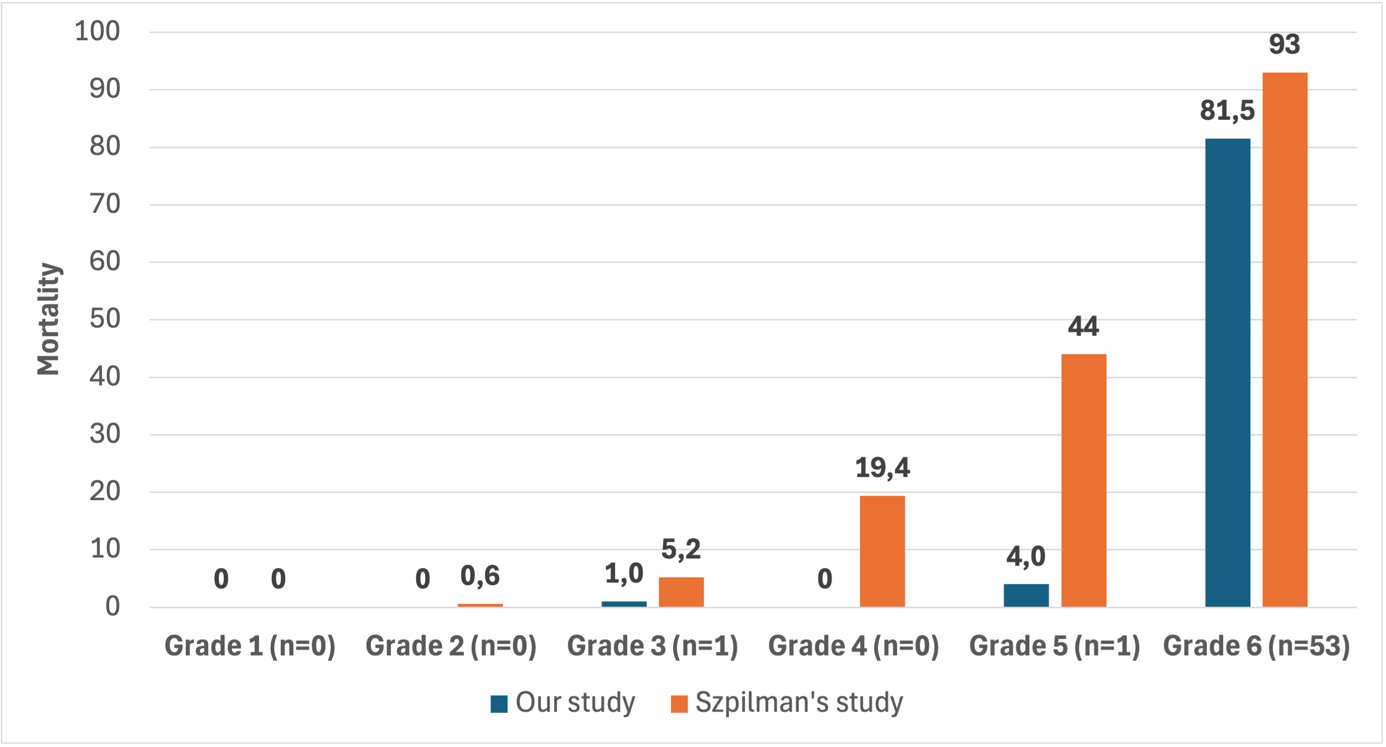


**Online supplement – 2.** Overall mortality by Szpilman classification: comparison between the VAR-Drowning cohort and the original Szpilman et al. cohort (Reference: Szpilman D. *Chest* 1997; 112(3): 660–5).

**Online supplement – 3.** Univariate analysis of prehospital predictive factors for ICU admission.

| **Variable** | **Observation** | **ICU admission** | **Class** | **Observation**  **N (%)** | **ICU admission**  **N (%)** | **OR [95% CI]** | **p-value** |
| --- | --- | --- | --- | --- | --- | --- | --- |
| **Age** – years | 270 | 130 | < 75 | 142 (53) | 58 (41) | Ref | - |
|  |  |  | ≥ 75 | 128 (47) | 72 (56) | 1.86 [1.15–3.02] | **0.012** |
| **Sex** | 270 | 130 | Male | 147 (54) | 65 (44) | Ref | - |
|  |  |  | Female | 123 (46) | 65 (53) | 1.41 [0.87–2.29] | **0.158** |
| **Previous chronic disease** | 251 | 124 | None | 62 (25) | 18 (29) | Ref | - |
|  |  |  | ≥ 1 | 189 (75) | 106 (56) | 3.12 [1.68–5.80] | **< 0.001** |
| **AVPU Scale** | 269 | 129 | A | 214 (80) | 79 (37) | Ref | - |
|  |  |  | V or P or U | 55 (20) | 50 (91) | 17.09 [6.54–44.64] | **< 0.001** |
| **HR** - bpm | 201 | 78 | < 100 | 118 (59) | 42 (36) | Ref | - |
|  |  |  | ≥ 100 | 83 (41) | 36 (43) | 1.39 [0.78–2.46] | **0.266** |
| **SBP** - mmHg | 205 | 82 | <100 | 8 (4) | 4 (50) | Ref | - |
|  |  |  | ≥ 100 | 197 (96) | 78 (40) | 0.66 [0.16–2.70] | **0.559** |
| **DBP** - mmHg | 150 | 72 | ≤ 60 | 20 (13) | 9 (45) | Ref | - |
|  |  |  | > 60 | 130 (87) | 63 (48) | 1.15 [0.45–2.96] | **0.774** |
| **SpO2** – % | 218 | 90 | ≥ 94 | 65 (30) | 6 (9) | Ref | - |
|  |  |  | < 94 | 153 (70) | 84 (55) | 11.97 [4.88–29.39] | **< 0.001** |
| **RR** – cycle per min | 135 | 80 | < 20 | 54 (40) | 25 (46) | Ref | - |
|  |  |  | ≥ 20 | 81 (60) | 55 (68) | 2.45 [1.21–4.99] | **0.013** |
| **Body temperature** - °C | 72 | 41 | ≥ 35 | 59 (82) | 33 (56) | Ref | - |
|  |  |  | < 35 | 13 (18) | 8 (62) | 1.26 [0.37–4.31] | **0.712** |
| **Witness drowning** | 265 | 128 | Lifeguard | 145 (55) | 62 (43) | Ref | - |
|  |  |  | Public | 120 (45) | 66 (55) | 1.64 [1.00–2.66] | **0.048** |
| **Immersion duration** - min | 220 | 99 | < 1 | 172 (78) | 56 (33) | Ref | - |
|  |  |  | ≥ 1 | 48 (22) | 43 (90) | 17.81 [6.69–47.44] | **< 0.001** |
| **Szpilman classification** | 270 | 130 | < 3 | 108 (40) | 123 (76) | Ref | - |
|  |  |  | ≥ 3 | 162 (60) | 56 (33) | 45.49 [19.51–106.05] | **< 0.001** |
| **Type of immersion** | 270 | 130 | Freshwater | 35 (13) | 17 (49) | Ref | **0.957** |
|  |  |  | Saltwater | 235 (87) | 113 (48) | 0.98 [0.48–2.00] |  |
| **Drowning in a lifeguards-supervised area** | 269 | 129 | Yes | 183 (68) | 82 (45) | Ref | **0.133** |
|  |  |  | No | 86 (32) | 47 (55) | 1.48 [0.89–2.48] |  |
| ICU: intensive care unit; A: alert; V: verbal; P: pain; U: unresponsive; HR: heart rate; SBP: systolic blood pressure; DBP: diastolic blood pressure; RR: respiratory rate. | | | | | | | |

**Online supplement – 4.** Multivariate analysis of prehospital predictive factors for ICU admission

| **Prehospital variable** | **Class** | **Observation**  **N (%)** | **ICU admission**  **N (%)** | **OR [95% CI]** | **p-value** |
| --- | --- | --- | --- | --- | --- |
| **Szpilman classification** | <3 | 96 (44) | 6 (6) | Ref | **-** |
|  | ≥3 | 123 (56) | 92 (75) | **29.85 [11.01–80.91]** | **< 0.001** |
| **AVPU scale** | A | 177 (81) | 60 (34) | Ref | **-** |
|  | V, P, or U | 42 (19) | 38 (90) | **4.37 [1.25–15.30]** | **0.021** |
| **Immersion duration** | <1 min | 172 (79) | 56 (33) | Ref | **-** |
|  | ≥1 min | 47 (21) | 42 (89) | **8.97 [2.50–32.19]** | **< 0.001** |
| ICU: intensive care unit; OR: Odds ratio; CI: confidence interval; AVPU: alert, verbal, pain, unresponsive. | | | | | |
